# Crosstalk Signaling Between the Epithelial and Non-Epithelial Compartments of the Mouse Inner Ear

**DOI:** 10.1007/s10162-025-00980-7

**Published:** 2025-03-13

**Authors:** Abel P. David, Sushobhan Biswas, Macey P. Soltis, Yasmin Eltawil, Ruiqi Zhou, Sarah A. Easow, Alan G. Cheng, Stefan Heller, Taha A. Jan

**Affiliations:** 1 Department of Otolaryngology – Head and Neck Surgery, Epithelial Biology Center, Vanderbilt Center for Stem Cell Biology, Vanderbilt University Medical Center, Preston Research Building, PRB 752, 2220 Pierce Ave, Nashville, TN 37232 USA; 2 Department of Otolaryngology – Head and Neck Surgery, Mass Eye and Ear, Harvard Medical School, Boston, MA 02114 USA; 3 Department of Otolaryngology – Head and Neck Surgery, Stanford University School of Medicine, Stanford, CA 94305 USA

**Keywords:** Inner ear, Epithelial-mesenchymal interaction, Utricle development, Hair cell, Periotic mesenchyme

## Abstract

**Purpose:**

The otolith organs of the inner ear consist of the utricle and saccule that detect linear acceleration. These organs rely on mechanosensitive hair cells for transduction of signals to the central nervous system. In the murine utricle, about half of the hair cells are born during the first postnatal week. Here, we wanted to explore the role and interaction of the non-epithelial mesenchymal cells with the sensory epithelium and provide a resource for the auditory neurosciences community.

**Methods:**

We utilized full-length Smart-seq2 single-cell RNA sequencing at postnatal days 4 and 6 along with a host of computational methods to infer interactions between the epithelial and non-epithelial compartments of the mouse utricle. We validated these findings using a combination of immunohistochemistry and quantitative multiplex in situ hybridization.

**Results:**

We report diverse cell–cell crosstalk among the 12 annotated cell populations (*n* = 955 cells) in the developing neonatal mouse utricle, including epithelial and non-epithelial cellular signaling. The mesenchymal cells are the dominant signal senders during the postnatal period. Epithelial to mesenchymal signaling, as well as mesenchymal to epithelial signaling, are quantitatively shown through the TGFβ and pleiotrophin pathways.

**Conclusion:**

This study highlights the dynamic process of postnatal vestibular organ development that relies not only on epithelial cells, but also on crosstalk between spatial compartments and among different cell groups. We further provide a data-rich resource for the inner ear community.

**Supplementary Information:**

The online version contains supplementary material available at 10.1007/s10162-025-00980-7.

## Introduction

The mammalian inner ear consists of five neurosensory vestibular organs and one auditory organ. The two otolith organs, the utricle and the saccule, detect linear acceleration in the horizontal and vertical planes, respectively. Hair cells are integral for the translation of mechanical stimuli during motion and from sound vibrations to electrical impulses that are delivered to the central nervous system [1]. The murine utricle is frequently used as a model to study the vestibular system because of its accessibility and unique postnatal feature of asynchronous development. It is an epithelial sheet with approximately 4000 hair cells interdigitated by supporting cells [2]. Following birth, the mouse utricle is functional yet continues its postnatal expansion of the sensory epithelium through the addition of both hair cells and supporting cells [2–4]. The stepwise molecular dynamics of cell addition to the sensory epithelium have been previously described [4, 5]. While epithelial and mesenchymal interactions have been studied in the cochlea during embryonic development [6–8], many investigations have neglected postnatal development of the inner ear with respect to the surrounding cell types that make up much of the inner ear organs.

Epithelial-mesenchymal interaction (EMI) during development has been well characterized in multiple organ systems including in the gastrointestinal tract, skin, mammary gland, and during tooth development. In these systems, crosstalk between the epithelial and mesenchymal cell compartments takes place. A signaling factor is produced by the epithelial cell compartment which elicits a response from the mesenchyme that in turn maintains a stem cell or induces a cellular function in the epithelium. Within the gastrointestinal tract, reciprocal EMI controls regionalization and development through signaling cascades that include Shh, Hox homeobox transcription factors, TGFβ signaling, BMP, Wnt, and forkhead transcription factors [9]. Homeostasis of the adult gastrointestinal tract also relies heavily on EMI, including in regeneration of intestinal epithelial stem cells [9]. In the skin, epithelial-mesenchymal interaction is spatially confined to micro-niches that coordinate multi-lineage tissue growth during hair follicle homeostasis [10]. EMI further plays a critical role during skin wound healing, where Wnt-β-catenin signaling increases collagen deposition in the extracellular matrix. Fibroblasts recruited to a skin wound have extensive Wnt, TGFβ, TNF, PDGF, and cytokine crosstalk with the epithelium [11, 12]. Both tooth development and regulation of dental stem cells utilize intricate EMI signaling networks. For example, epithelial Fgf9 regulates mesenchymal FGF signaling to maintain epithelial dental stem cells [13]. Likewise, EMIs are integral to the development and homeostasis of the mammary gland at different stages, such as embryonic cell specification, pubertal growth, pregnancy, lactation, and involution. During development, mesenchymal cells provide inductive signals to the overlying epithelium to sequentially restrict epithelial cell fates along the mammary lineage [14]. Development of the mammary gland involves parathyroid hormone-related protein that is produced by the mammary epithelium and acts on the mesenchymal cells to modulate Wnt and BMP signaling cascades.

These systems illustrate the intricate relationships and reciprocal crosstalk between epithelial and mesenchymal compartments. They involve some of the canonical morphogens during embryonic development, adult homeostasis, and increasingly during injury/repair mechanisms. In the cochlea, for example, developmental influences of the canonical Wnt pathway have been studied between the periotic mesenchyme and the sensory epithelia [15]. Wnt-activated periotic mesenchymal cells take on an epithelial phenotype, while Wnt-activated cochlear epithelial cells dedifferentiate and lose epithelial markers. Separately, cochlear progenitor cell numbers have been shown to be regulated by mesenchymal FGF receptor signaling [8]. The ETS-domain transcription factors ETV4 and ETV5 have recently been shown to be downstream of FGF signaling within the mesenchyme [6]. Furthermore, the identity and function of the surrounding periotic mesenchyme and other cell types of the inner ear is clinically important with multiple genes that cause hearing loss without affecting the sensory epithelium directly [16, 17]. While other investigations have identified embryonic and postnatal cell types of the periotic mesenchyme in the crista and cochlea, the utricle and its postnatal development with respect to cellular crosstalk remain unelucidated [17, 18]. In this work, we detail the transcriptional heterogeneity of the mouse utricle at postnatal day (P)4 and P6 during expansion of the sensory epithelium. We use a variety of computational and validation techniques to demonstrate the critical relationships among different cell compartments and cell types within the inner ear. Importantly, we provide a high-quality full-length Smart-seq2 sequenced single-cell RNA-seq dataset for the auditory neuroscience community.

## Methods and Materials

### Animals

All mice used for validation experiments were wild-type FVB (JAX #001800) obtained from the Jackson Laboratory. Non-epithelial compartment cellular transcriptomic data was obtained from P4 and P6 wild-type FVB and *Pou4f3*^*DTR/*+^ mice (Jackson Laboratory, #028673 and #029802). The *Pou4f3*^*DTR/*+^ transgene was not activated, similar to our previous study [4]. Epithelial transcriptomic data was obtained from our publicly available dataset from P4 and P6 mice which also used FVB and *Pou4f3*^*DTR/*+^ mice [4]. Mice were kept in a controlled environment with 12-h light–dark cycles under the care of Stanford Veterinary Service Center or the Vanderbilt Animal Care and Use Program, both accredited by the AAALAC. Institutional protocol approvals were obtained (Protocol #18606 Stanford University; #M2200033 Vanderbilt University). Mice of both sexes were used in this study. We have attempted to abide by the ARRIVE guidelines throughout the manuscript [19].

### Single-Cell RNA-Seq Tissue Preparation

Mice were sacrificed at indicated ages according to approved institutional protocols. We used our previously described protocol [4]. Briefly, utricles were isolated in ice-cold HBSS. Samples were then pooled from multiple animals (3–4 mice) and incubated with thermolysin (0.5 mg/mL; Sigma T7902) for 20 min at 37 °C. The sensory epithelium was peeled away from the mesenchyme mechanically. Pooled samples were incubated with Accutase (ThermoFisher 00–4555-56) for 6 min at 37 °C. Single-cell suspension was achieved by gentle trituration 40 times using a p1000 pipette with subsequent filtering using a 40-μm filter. As previously described, cells were FACS sorted (Sony SH800) into 96-well plates following the addition of propidium iodide (PI, BD Biosciences, 556463) for cell death exclusion [4]. Doublets were gated out using size exclusion. Cells were sorted directly into RNase inhibitor, 0.1% Triton X-100, dNTPs, and oligodT30VN prior to freezing at -80 °C. A total of 12 mice (both sexes) were used in this study for the non-epithelial cells (4 pups were P4 and 8 pups were P6). This resulted in seven 96-well plates for sorting. This protocol is the same as our previous study where the sensory epithelial cells were collected [4].

### Library Prep and Sequencing

96-well plates were submitted to the Stanford Functional Genomics Facility (SFGF) for processing using the Smart-seq2 protocol [20]. Each cell was barcoded and reverse transcribed. cDNA was assessed for each single cell using an Agilent 2100 Bioanalyzer prior to selection of cells for library prep. Cell libraries were then sequenced using an Illumina NextSeq500 with a goal of 1 million reads per cell using 75 bp paired-end full length reads. Following barcode demultiplexing, FASTQ files were obtained from SFGF through the local Sherlock High Performance Computing cluster. This was the same procedure as our previous study [4].

### Data Processing

FASTQ files were aligned using *STAR* aligner (v2.5.3a) to the mouse genome (GRCm38 mm10) and quantified using *RSEM* (v1.3.0) [21, 22]. MultiQC was used to assess read quality [23]. Generated count matrices were imported into R statistical computing environment for downstream analysis.

### Quality Control and Normalization

We utilized previously published data for all sensory epithelial samples [4]. This included 1002 unfiltered cells that were downloaded from NCBI (GSE155966). The previously published data contained cells from ages P2, P4, and P6. Here, we only utilized the P4 and P6 cells from the previously published data. There were a total of 471 non-epithelial cells (new from this study) and 684 epithelial cells (from our prior study [4]) before quality control. ERCC spike-ins and mitochondrial gene percentages were calculated. All cells expressing more than 10% ERCC spike-in and containing more than 10% mitochondrial genes were excluded. Then, the isOutlier() function from *scuttle* was used to filter both outlier read counts (library size) and outlier number of detected features (genes). Those with a median absolute deviation (MAD) of 3 or greater were then filtered out. Gene levels were parsed by removing all genes that were not expressed in at least 3 cells, leaving a total of 25,695 genes. All data was processed using *Seurat*’s standard pipeline. Following quality control, we had a total of 955 cells comprised of high-quality epithelial and non-epithelial tissues. This was composed of 312 P4 cells and 643 P6 cells. Normalization was performed using the *Seurat* NormalizeData() function.

### Cell Clustering and Differential Gene Expression

We utilized the *Seurat* package to perform standard data pre-processing steps, including normalization, scaling, dimension reduction, and clustering. Uniform manifold approximation and projection (UMAP) dimensionality reduction was used. Graph-based clustering was then performed, cells were first embedded in a K-nearest neighbor (KNN) graph structure, and then a Louvain clustering algorithm was applied using the functions FindNeighbors() and FindClusters() using a clustering resolution of 0.8. The clustering resolution was determined using the *ChooseR* package, and an optimal resolution of 0.8 was determined to minimize over-clustering and over-splitting [24]. Differential gene expression was then performed to identify the highest differentially expressed genes among the different cell clusters with a log_2_ fold change of 2 or greater using the Wilcoxon rank sum test. The adjusted *p*-values are based on the Bonferroni correction method using all genes in the dataset using the function FindAllMarkers().

### Comparative Analysis of Cochlea and Utricle

We used the publicly available dataset from Rose et al. available at NCBI (GEO GSE217727) [17]. The P7 cochlear dataset was downloaded and preprocessed using the previously described thresholds for quality control in *Seurat *[17]. Following doublet removal using *DoubletFinder*, a UMAP was generated with a total of 4256 cochlear mesenchyme cells. The four subtypes of cochlear mesenchyme were annotated using marker genes from the published paper. Next, we subsetted out our utricle mesenchyme cells (P4 and P6) and created a merged *Seurat* object that subsequently underwent canonical correlation analysis (CCA) integration using *Seurat *[25]. Briefly, CCA integration takes the two datasets and harmonizes them by identifying anchoring genes following principal component analysis [25]. Notably, CCA integration has successfully been used for integrating single-cell RNA-seq datasets across batches, sequencing platforms, and single-cell data modalities. Specifically, it can integrate 10X and Smart-seq datasets [25]. Following integration, the entire dataset comprised of the utricle mesenchyme and cochlea mesenchyme underwent dimension reduction and plotted as a UMAP (Fig. S2C). To assign the utricle cells from the same embedding to the four subtypes of cochlear mesenchyme, we calculated the Euclidian distance for each utricular cell to the centroid of the four cochlear mesenchyme clusters which we considered as the reference. A summary table in R was then generated and plotted as a sunburst plot in Excel (Fig. S2D).

### Cell–Cell Communication Analysis

*CellChat* was used to infer the cellular communication patterns present in the dataset [26]. The specific ligand-receptor database used is maintained through *CellChatDB*, and the mouse species library consists of 2021 validated molecular interactions including 60% secreted autocrine/paracrine signaling interactions, 21% extracellular matrix (ECM)-receptor interactions, and 19% cell–cell interactions. Expression data was pre-processed using a standard workflow. Cellular communication patterns were visualized using the available methods: netVisual_circle(), netAnalysis_contribution(), netVisual_bubble(), and plotGeneExpression(). Signaling roles were identified as senders, receivers, mediators, and influencers through the calculation of network centrality scores. These network analysis scores were visualized using netAnalysis_signaling() and Role_network().

### Data Availability

The raw sequencing data, including all FASTQ files and metadata, is deposited in the NCBI Gene Expression Omnibus (GSE279713). Data is also uploaded into gEAR (https://umgear.org/p?l=025ab571), which is a publicly available portal for visualization and analysis of multiomic data.

### Code Availability

The code used to generate figures and plots is from standard indicated R package manuals. We have also created a detailed and annotated GitHub repository that contains all code used for the analysis described and to generate the figures (https://github.com/tahajanlab/published).

### Immunohistochemistry

Whole mount utricles were prepared and processed as previously described [4, 27]. Ice-cold HBSS was used for microdissection of utricles out of the temporal bone followed by 4% paraformaldehyde (Electron Microscopy Sciences 15710) fixation for 40 min on ice. Triton X-100 (0.1%) in PBS was used to wash the samples for 15–30 min three times. Blocking for non-specific antigens was performed using 5% donkey serum, 0.1% Triton X-100, 1% BSA (ThermoFisher BP1600), and 0.02% sodium azide (Sigma, S2002). Specimens were incubated with blocking solution at room temperature for 30–60 min prior to application of indicated antibodies overnight at 4 °C. The following day, samples were washed with 0.1% Triton X-100 in PBS three times, and secondary antibody was added with DAPI for 2 h at room temperature. Samples were once again washed with PBS alone three times prior to mounting using ProLong Gold Antifade mounting media (Invitrogen P36930). All experiments were performed in triplicate unless otherwise stated. Images were acquired using an A1R confocal microscope or Zeiss Apotome 3 upright system. Figures were prepared using Adobe Photoshop and Illustrator. Primary antibodies used in this study include rabbit-anti-Myosin7a (1:500–1:1000, Proteus Biosciences, 25–6790), monoclonal mouse-anti-Cytokeratin18 (1:200, Proteintech, 66187–1-Ig), rabbit-anti-Iba1 (1:1000, FUJIFILM Wako Chemicals, 019–19741), rat-anti-Pecam1 (1:50, Fisher, 551262), and mouse-anti-Epcam (1:1000, BioLegend, 118201). All secondary antibodies were Invitrogen Alexa Fluor® used at 1:400.

### *In Situ* Hybridization

*RNAscope*^*TM*^ from Advanced Cell Diagnostics was used for all in situ hybridization experiments. For cryosectioning, the protocol established by Jansson et al. (2018) [28] was followed. Briefly, otic capsules were fixed in 10% neutral buffered formalin for 12–16 h at 4 °C prior to serial sucrose gradient exposure and mounting in optimum cutting temperature (OCT) compound over dry ice. Tissue was sectioned at 10 microns 1–3 days prior to *RNAscope*^*TM*^ protocol. *RNAscope*^*TM*^ commercial protocols were used for multiplex fluorescent probe detection. Co-labeling with antibodies was performed at the very end of the protocol following the last amplification step. Antibodies were incubated with the tissue at indicated concentrations overnight at 4 °C in 0.1% Triton X-100. The next day, secondary antibody and DAPI incubation were performed as detailed above in the Immunohistochemistry section. All *RNAscope*^*TM*^ experiments were performed in triplicate unless otherwise stated.

### RNAScope^TM^ Quantification

For quantification of in situ hybridization (*RNAscope*^*TM*^), images were taken on a Nikon A1R confocal microscope and imported into ImageJ2 (Fiji, v2.14.0/1.54f) image processing software. Within Fiji, 6 consecutive z-stack slices were flattened into one image within a Z project. The number of condensed slices was kept consistent at 6 among each quantified image. While referencing the Myosin7a and DAPI fluorescent signal of the original composite image, a polygon was drawn around the mesenchymal (MES) and separately, the sensory epithelial (SE) compartments. Regions of background were excluded. These representative areas were then renamed and saved into the region of interest (ROI) manager to be utilized further downstream. On the single-color channel image, the background was subtracted with the set parameter of 3.0 pixels, and the threshold was taken with the given settings in Otsu applied. Steps to allow the image to be analyzed were then taken by making the image binary and then converting it to a mask. The image was further processed by selecting “close” and “watershed” in the binary tab. Lastly, the number of puncta was quantified with the saved areas in the ROI manager. This was done using the “Analyze Particles” function in Fiji to auto-count all of the puncta within the defined SE field and separately within the MES field. The MES field and SE field areas were then quantified using Fiji’s measure area tool (Command + M). The puncta counts and the whole area of the MES and SE fields were then recorded. If any red blood cell background was present within either the SE or MES, their area and count numbers were recorded and subtracted from their corresponding area. The respective number of puncta per MES or SE area was then calculated and plotted as a violin plot. In this way, we achieved the number of puncta per measured area to account for any differences in sectioning. Quantification violin plots and *t*-tests were performed in R statistical computing environment.

## Results

### Heterogeneity of Epithelial Cells

**Fig. 1:**
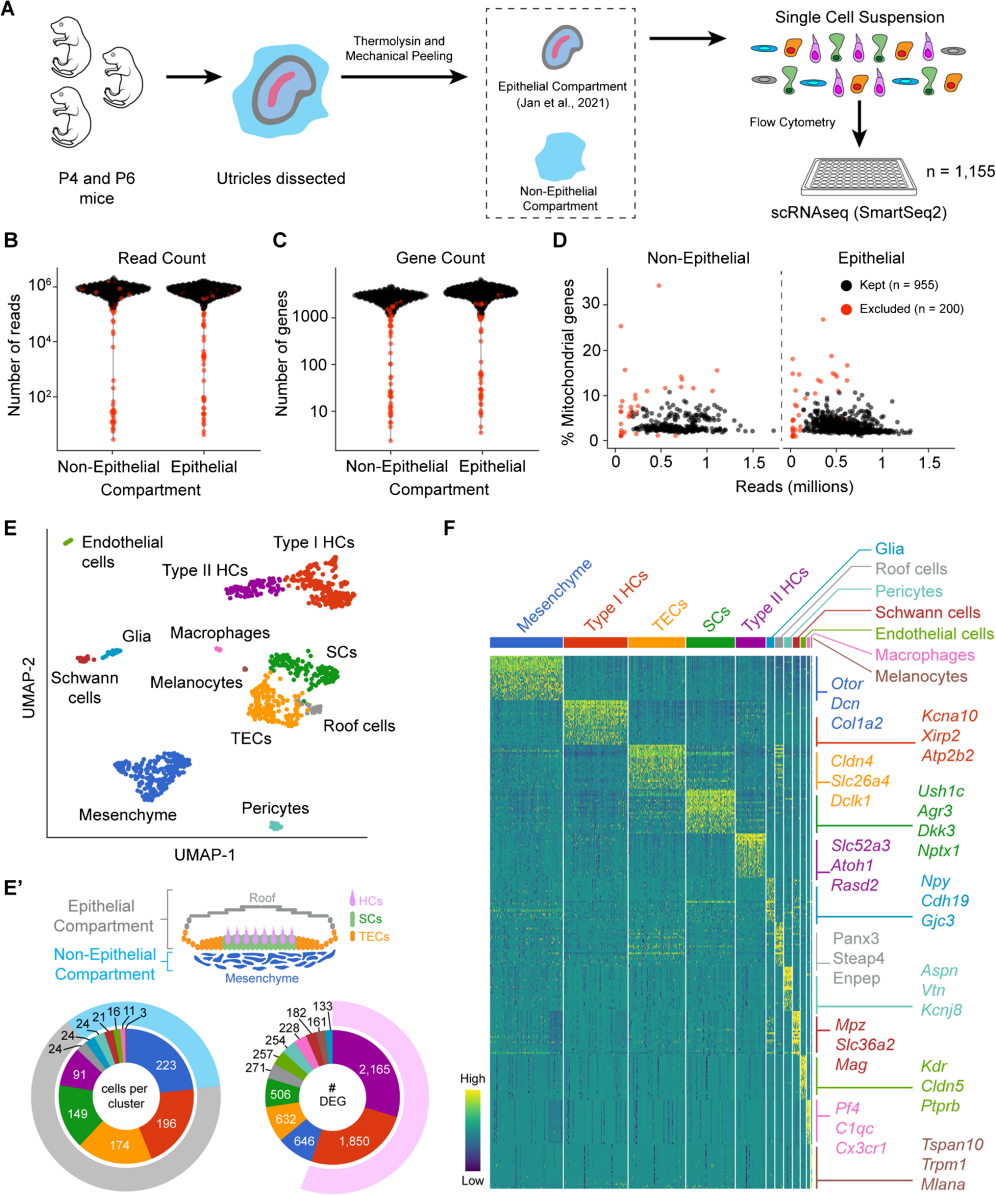
Transcriptomic diversity of the postnatal mouse utricle. **A** Schematic illustration of experimental setup depicting the harvest of utricles from neonatal mice (age P4 and P6). The sensory epithelium was separated from the underlying non-epithelial tissues following enzymatic digestion with thermolysin and mechanical peeling. These two compartments were separately placed into a single-cell suspension and underwent plate-based single-cell RNA sequencing using the Smart-seq2 method. Quality control was performed, and the cells that were excluded are highlighted in red and were based on **B** low read counts, **C** low gene counts, and **D** high percentage of mitochondrial genes. **E** UMAP plot of all cells (*n* = 955) that passed quality control. There were twelve identified clusters, and marker genes were used to annotate the cell types. **E’** Cross-sectional diagram of the utricle highlighting the epithelial and non-epithelial cell compartments. The sunburst plot on the left shows the number of cells per cluster with the outer gray rim representing the epithelial compartment and the light blue rim representing the non-epithelial compartment. The sunburst plot on the right depicts the total number of differentially expressed genes per cell group, highlighting the two hair cell groups in light purple as the cells with the highest number of differentially expressed genes. **F** Heatmap showing the top 25 differentially expressed genes in each cell cluster with representative genes shown on the right side of the heatmap (full list is provided in Supplementary Data File 1). The color on the heatmap depicts relative expression from low (dark blue) to high (yellow) expression

To investigate the epithelial and non-epithelial compartments of the utricle, we first mechanically separated these compartments (Fig. 1A), allowing for individual cell captures. We have previously characterized the epithelial cells of the postnatal mouse utricle [4]. Here, we used our previously published data from P4 and P6 sensory epithelia and added newly acquired P4 and P6 non-epithelial cells using the Smart-seq2 protocol [29]. Using a series of quality control steps (Fig. 1B–D), we identified 329 non-epithelial cells and 626 epithelial cells. The median read count for non-epithelial cells is 625,900, and for epithelial cells is 583,033. The median number of genes detected per cell was 2672 and 3326.5 in non-epithelial and epithelial cells, respectively. We were curious by these differences in detectable genes per cellular compartment and found that among the 12 subsequently annotated distinct cell groups (Fig. 1E), the median number of detectable genes per cellular compartment loosely correlated with the cell type’s differentiation state (Fig. 1E’). In other words, taking the number of differentially expressed genes as a surrogate of differentiation status, we observe that hair cells account for most of the difference between the median number of detectable genes between the epithelial and non-epithelial cell compartments (Fig. 1E’). Differential gene expression using a non-parametric Wilcoxon rank sum test showed a total of 16,611 genes with a false discovery rate (FDR) < 0.1 (Fig. 1F). Cell annotation based on known marker genes revealed five epithelial cell types and seven non-epithelial cell types. Similar to our prior findings, the epithelial cells fall into categories of type I (*Xirp2*, *Kcna10*, *Atp2b2*) and type II (*Slc52a3*, *Atoh1*, *Rasd2*) hair cells, supporting cells (*Ush1c*, *Dkk3*, *Nptx1*, *Agr3*), transitional epithelial cells (TECs) (*Dclk1*, *Slc26a4*, *Cldn4*), and roof cells (*Enpep*, *Steap4*, *Panx3*). The non-epithelial cells are comprised of periotic mesenchyme (*Otor*, *Dcn*, *Col1a2*), pericytes (*Vtn*, *Aspn*, *Kcnj8*), Schwann cells (*Mpz*, *Mag*, *Slc36a2*), glia (*Plp1*, *Cdh19*, *Gjc3*), endothelial cells (*Kdr*, *Cldn5*, *Ptprb*), macrophages (*Cx3cr1*, *C1qc*, *Pf4*), and melanocytes (*Tspan10*, *Mlana*, *Trpm1*). Taken together, these results define the cellular heterogeneity of the utricle with the epithelial compartment comprised of the hair cells, supporting cells, TECs, and roof cells, while the non-epithelial compartment is comprised primarily of mesenchyme with distinct other cell types.

### Epithelial and Non-Epithelial Cell Compartments

**Fig. 2:**
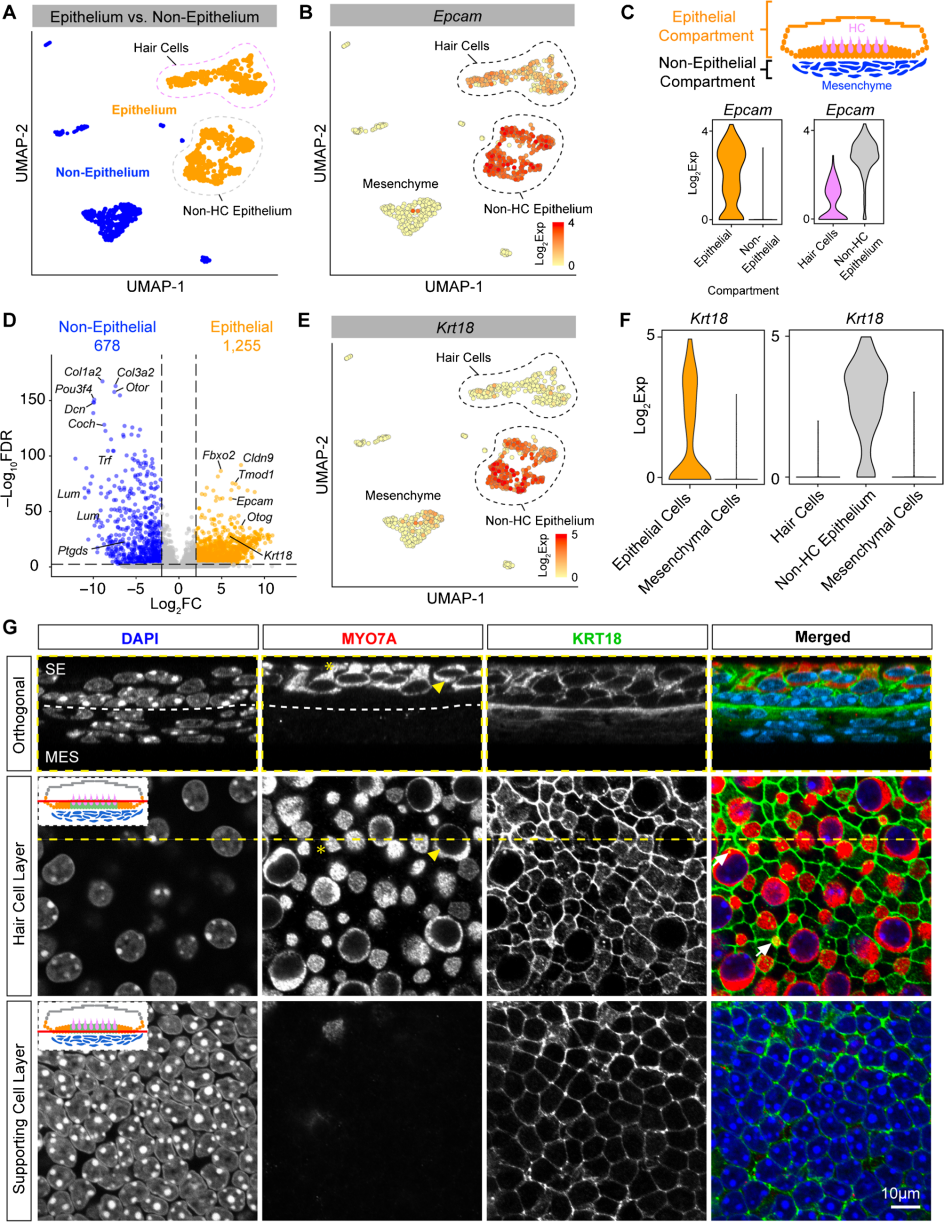
Epithelial and non-epithelial compartments of the utricle. **A** UMAP plot depicting the sensory epithelial cells in orange and their hair cell and non-hair cell subsets and the non-epithelial cells in blue. **B** *Epcam* expression plot demonstrating the log2 expression in each cell plotted on the UMAP. **C** *Epcam* is a marker that differentiates the epithelial compartment from the non-epithelial compartment and is present in both hair cells and non-hair cell epithelium, albeit lower in expression in hair cells. **D** Volcano plot demonstrating the genes enriched in the epithelial cells versus the non-epithelial cells as calculated by pseuodbulk *DESeq2* analysis. A total of 678 genes are enriched in the non-epithelial compartment, while 1255 genes are enriched in the epithelial compartment (full list of genes provided in Supplementary Data File 2). **E, F** *Krt18* is a marker that is found primarily in non-hair cell epithelial cells as shown on this UMAP expression plot and violin plot with occasional low expression in hair cells. **G** Anti-KRT18 antibody stains primarily the supporting cells (green) in the epithelial cell layer, with anti-MYO7A (red) as a hair cell cytoplasmic stain and nuclei stained with DAPI (blue). Orthogonal image is at the level of the hair cell layer indicated by the yellow dotted line. There are occasional hair cells that co-label with MYO7A (white arrows in merged column). Note the lack of expression of KRT18 in the mesenchyme layer. Cartoon diagram inset depicts (red horizontal line) the level at which the image was acquired. SE, sensory epithelium; MES, mesenchyme. Asterisks and arrowheads indicate the same cells

We first focused our analysis on defining the differentiating features between the epithelial and non-epithelial compartments of the utricle (Fig. 2A). Expression of the marker gene Epcam (CD326) has previously been reported as a pan-epithelial marker (Fig. 2B) [30]. This validated our metadata and computational prediction that hair cells, supporting cells, transitional epithelial cells, and roof cells are epithelial in origin. Notably, *Epcam* expression is lower in hair cells (type I and type II hair cells) as a group compared to the remainder of the epithelial cells (Fig. 2B and C). Direct comparison of non-epithelial versus epithelial cells with a non-parametric Wilcoxon rank sum test shows 678 genes to be differentially enriched in the non-epithelial cells and 1255 genes differentially enriched in the epithelial cells (Fig. 2D). To validate these computational results, we selected Krt18 for immunohistologic analysis. We chose *Krt18* as it is a well-known epithelial marker in other systems but has not been characterized in the inner ear. *Krt18* is more highly expressed in epithelial cells compared to mesenchyme (Fig. 2D–F) (*p* < 0.001). Antibodies to Krt18 label cells in the epithelial cell layer, primarily supporting cells, with some limited expression in hair cells (Fig. 2G, Fig. S1).

### Perivascular and Immune Cells

**Fig. 3:**
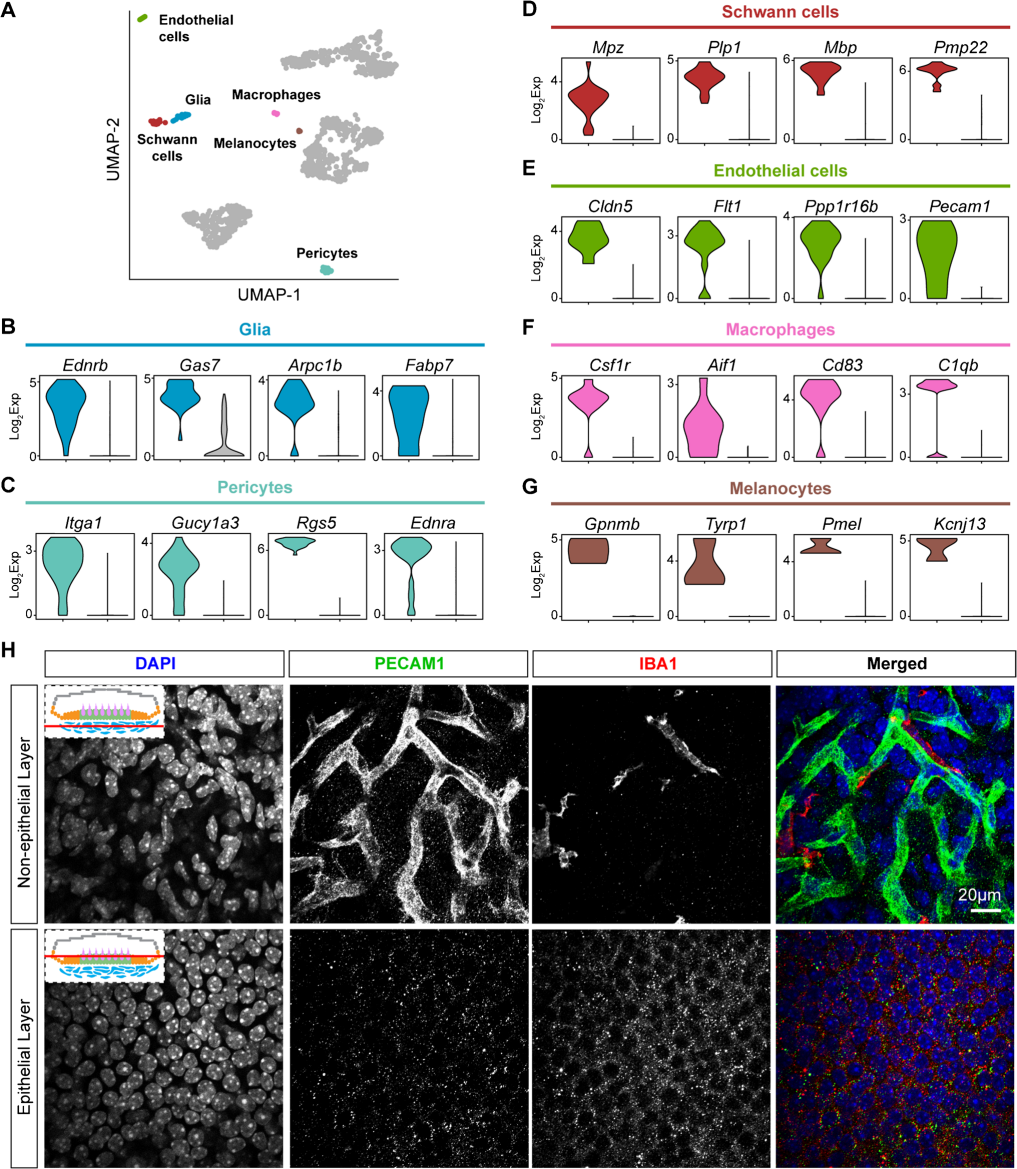
Non-mesenchymal cell types of the non-epithelial compartment. **A** UMAP highlighting annotated non-epithelial and non-mesenchymal cell clusters. **B**–**G** Violin plots of four differentially expressed genes in each cell cluster compared to all other cells in the dataset on a log2 expression scale. **H** Whole mount immunohistochemistry of the mouse utricle showing the macrophage marker anti-IBA1 (red, also known as AIF1) and the endothelial cell marker anti-PECAM1 (green) found adjacent to each other in the non-epithelial compartment. These two cell markers are absent in the epithelial compartment (second row). Cartoon diagram inset depicts (red horizontal line) the level at which the image was acquired

The non-epithelial compartment of the utricle is rich in other cell types: endothelial cells, pericytes, Schwann cells, glia, macrophages, and melanocytes. Each of these cell groups has unique molecular markers when compared to all other cells in the dataset (Fig. 3A–G). Glial cells express marker genes such as *Ednrb*, *Arpc1b*, and *Fabp7* (Fig. 3B). Intimately associated with the vasculature are pericytes that express *Itga1*, *Gucy1a3*, *Rgs5*, and *Ednra* (Fig. 3C). *Mpz*, *Plp1*, *Mbp*, and *Pmp22* are all known Schwann cell genes that are defining features of utricular Schwann cells as well (Fig. 3D) [16]. The vasculature of the utricle consists of vascular endothelial cells that express *Cldn5*, *Flt1*, *Ppp1r16b*, and *Pecam1* (Fig. 3E). Utricle macrophages express hallmark genes such as *Csf1r*, *Aif1*, *Cd83*, and *C1qb* (Fig. 3F) [31]. Finally, melanocytes are pigment-expressing cells of the inner ear that display *Gpnmb*, *Tyrp1*, *Pmel*, and *Kcnj13* (Fig. 3G). The macrophage marker, Iba1 (*Aif1*), is observed to be adjacent to the rich vasculature of the non-epithelial compartment as marked by Pecam1 immunolabeling and absent from the epithelial compartment (Fig. 3H). These diverse cell types highlight the non-epithelial compartment’s roles in nutrient supply via the vasculature and the prominent role of the immune system via macrophages and glial cells.

### Mesenchymal Cells

**Fig. 4:**
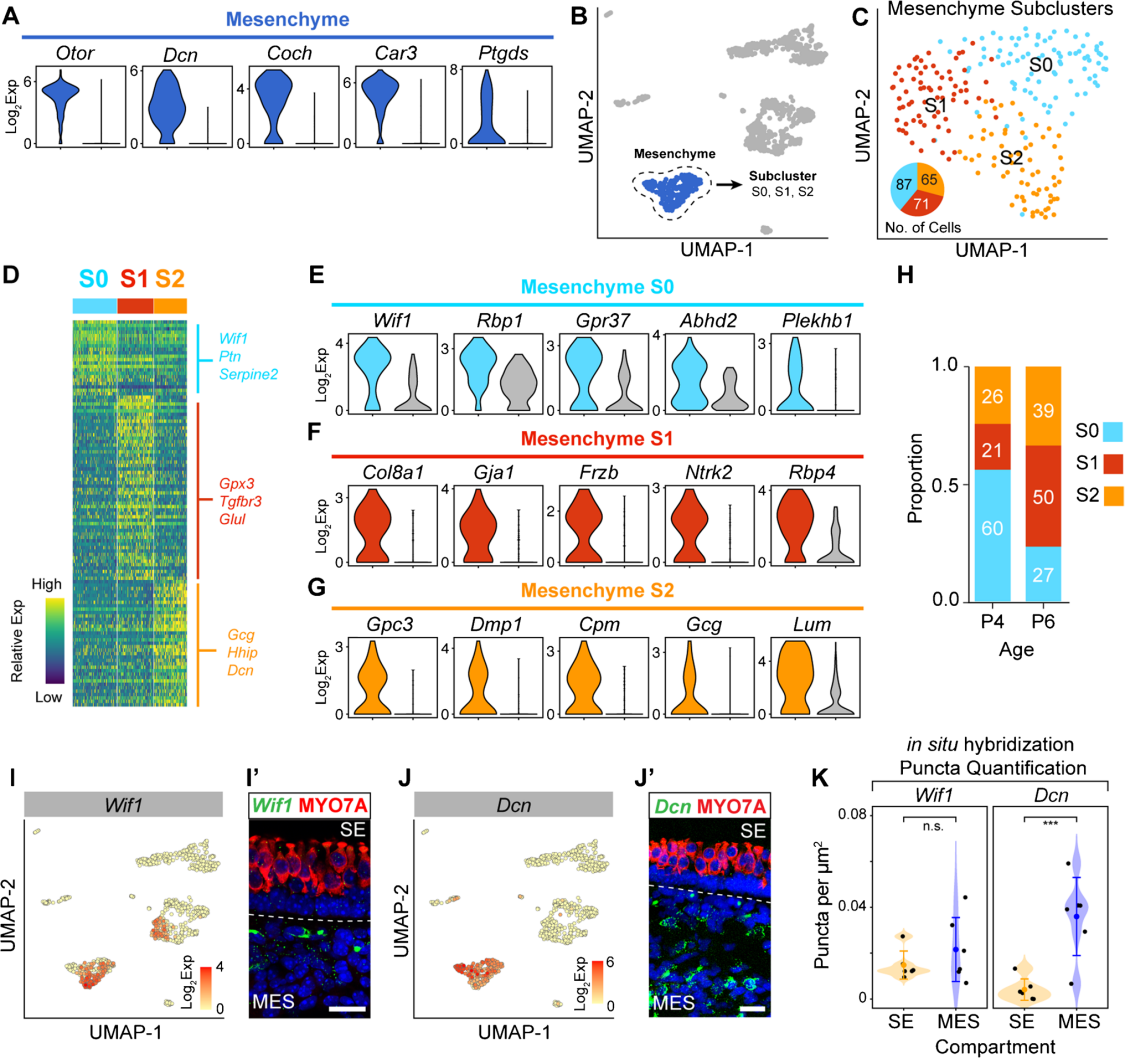
Heterogeneity of mesenchymal cells. **A** The top differentially expressed genes in the mesenchymal cell cluster compared to all other cells plotted on a log2 expression scale. **B** UMAP highlighting the mesenchyme cell cluster in blue, and these cells were subsetted and reclustered. **C** Reclustering of the mesenchyme cell group revealed three computationally distinct cell states (S0, S1, and S2) and are plotted on a UMAP, with their distribution in the pie chart inset. **D** Heatmap of the top differentially expressed genes in each mesenchymal cell state (subcluster) with the top three highlighted genes listed on the right (complete list of genes in Supplementary Data File 3). **E**–**G** Violin plots depicting the log2 expression of the representative differentially expressed genes in the three different cell states determined by reclustering the mesenchymal cell group, **E** for S0, **F** S1, and **G** S2. **H** The proportion of cells originating from P4 and P6 mice in each cell state are plotted here. In S0, there is a statistically greater proportion of P4 origin cells when compared to S1 and S2 states based on *X*^*2*^-testing (*X*^*2*^(2, *n* = 223) = 26.642, *p* < 0.001). **I, I’** UMAP plot showing log2 expression of the mesenchymal marker *Wif1*. Combined in situ hybridization of *Wif1* and immunohistochemistry of MYO7A shows *Wif1* mRNA (green) to be primarily localized to the mesenchymal layer (this is a part of the full image shown in Fig. S2D). DAPI is shown in blue. **J, J’** UMAP plot showing *Dcn* expression to be highly specific to mesenchymal cells with combined in situ hybridization and immunohistochemistry validation confirming expression of *Dcn* mRNA in the mesenchymal layer (this is part of the full image shown in Fig. S2E). DAPI is shown in blue. **K** Quantification of in situ hybridization puncta shows that *Wif1* expression trends towards higher levels in the mesenchyme, although this is not statistically significant (*t*-test, *p* = 0.3232), while expression of *Dcn* is much higher in the mesenchymal compartment compared to the sensory epithelium (*t*-test, = 0.00525). Scale bars = 20 µm; SE, sensory epithelium; MES, mesenchyme

The mesenchymal cells within the non-epithelial compartment are the dominant cell type (Fig. 1E). The main cluster of mesenchymal cells is characterized by the expression of *Otor*, *Dcn*, *Coch*, *Car3*, and *Ptgds* (Fig. 4A). Within the cochlea, there are multiple types of mesenchymal cells during development prior to the onset of hearing [17]. We therefore hypothesized that there are also different subgroups of mesenchymal cells within our data. To answer this question, we subset the mesenchymal cells into a separate manifold (Fig. 4B and C). Reclustering revealed three distinct subpopulations of mesenchyme (S0, S1, and S2) (Fig. 4C). Differential gene expression among these three clusters shows unique marker genes such as *Plekhb1*, *Col8a1*, and *Dmp1* for S0, S1, and S2, respectively (Fig. 4D–G). We further hypothesized that some of the clusters of mesenchymal cells are age-dependent. To test this, we determined the proportion of cells of each cell cluster based on age (Fig. 4H). This demonstrates that in cluster S0, there is a higher proportion of cells from P4 than P6 when compared to clusters S1 and S2 (Chi-squared, *p* = 1.639 × 10^−6^).

While some of the pan-mesenchymal markers, such as *Ptgds*, are ubiquitous in the mesenchyme of multiple organs, others are specific to the inner ear, such as *Otor* and *Coch* [32, 33], highlighting the unique role of mesenchyme within the inner ear. We performed validation experiments in postnatal utricles focusing on *Wif1* and *Dcn* expression (Fig. 4I–K, S2A–B), both of which are pan-mesenchymal marker genes. Combined in situ hybridization with anti-MYO7A immunohistochemistry shows that *Wif1* mRNA is localized primarily in the non-epithelial compartment of the utricle (Fig. 4I and I’; S2A). *Dcn*, another mesenchymal marker, was also primarily localized to the mesenchymal layer (Fig. 4J and J’; S2B). Given the quantitative nature of the transcriptomic computational analysis, we sought to determine if this translated to the tissue level. We therefore quantified mRNA expression in our tissue sections for both *Wif1* and *Dcn*. Quantification of mRNA puncta was carried out and separated by epithelial and non-epithelial compartments (Fig. 4K). There is a statistically significant difference between *Dcn* expression in the epithelial compartment compared to the non-epithelial compartment. However, analysis of *Wif1* compartments showed that while there is a trend toward high expression in the non-epithelial compartments, this is not statistically significant (Fig. 4K). One possible explanation for the variability seen with *Wif1* in the non-epithelial compartment may be hidden spatial distributions that are not well captured by cryosections and better determined on whole mount preparations.

We next compared the utricular mesenchymal cells with recently investigated cochlear periotic mesenchyme. Rose et al. (2023) showed that the developing cochlea from embryonic day 15 and postnatal day 7 harbors four spatially distinct mesenchymal cell populations consisting of basement membrane (type I), spiral limbus (type II), modiolar (type III), and lateral wall (type IV) [17]. We reasoned that our postnatal dataset from P4 and P6 is closest developmentally to the available cochlear postnatal P7 dataset from Rose et al. (2023). Therefore, we integrated the P7 cochlea mesenchyme data with our P4 and P6 utricle mesenchyme data into the same manifold UMAP space (Fig. S2C). The utricle mesenchyme transcriptionally resembles the type IV lateral wall and type II spiral limbus cochlear mesenchyme. Nearly 90% of the utricular mesenchyme co-cluster with the type IV and type II cochlear mesenchyme (Fig. S2D). These findings support the notion that the mesenchymal cells of the vestibular apparatus resemble the cochlear mesenchyme.

### Crosstalk Between Cell Groups

**Fig. 5:**
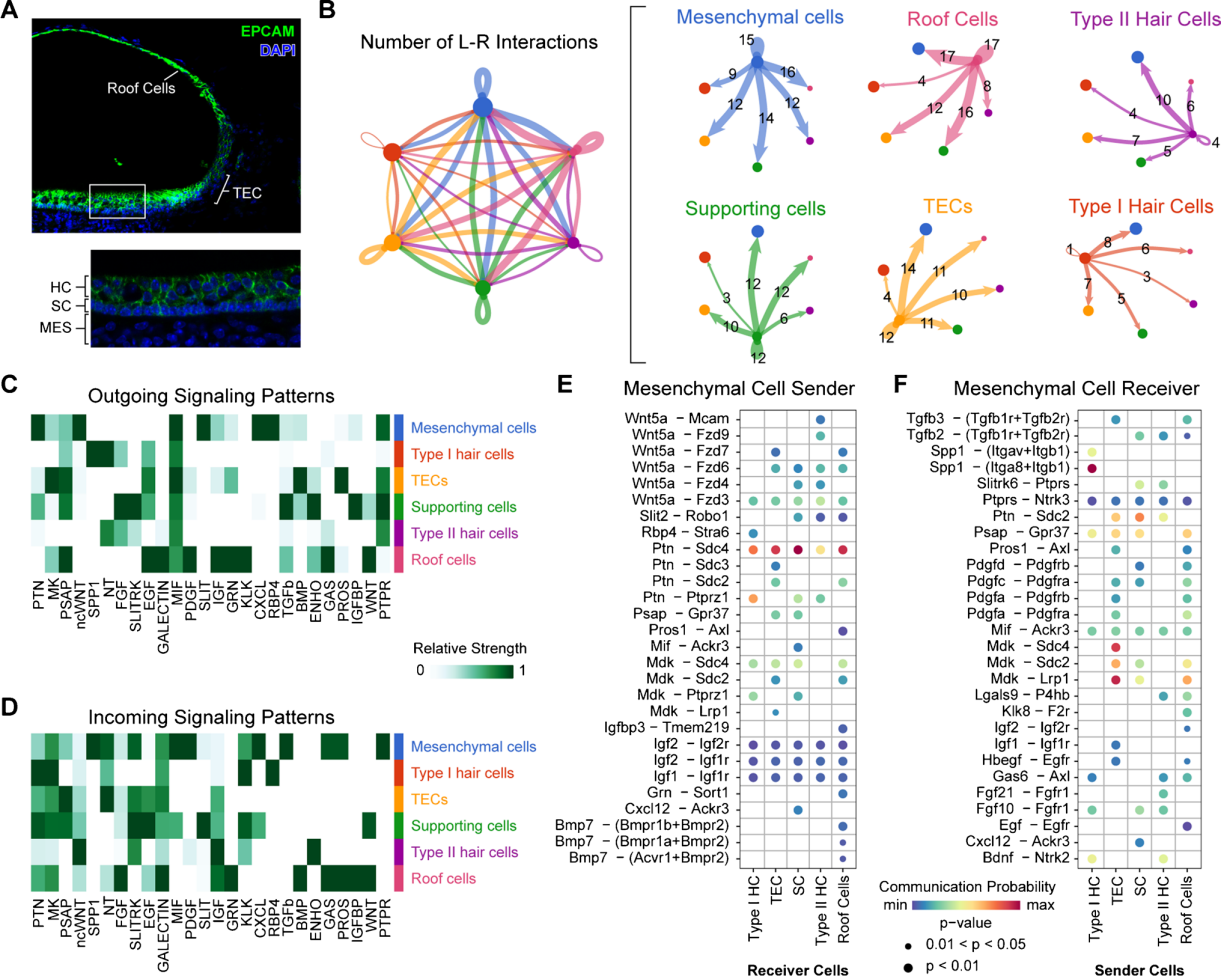
Mesenchymal cells are the dominant signal senders. **A** A confocal microscopy image staining epithelial cells marked by anti-EPCAM antibody (green) and DAPI nuclei (blue) showing the location of roof cells, transitional epithelial cells (TEC), hair cells (HC), and supporting cells (SC). The mesenchymal (MES) cell compartment is located beneath the epithelial cells. **B** A plot depicting the number of inferred ligand-receptor interactions between each cell cluster. Individual outgoing patterns for each cell type are shown, and the thickness of each arrow represents the number of paracrine signaling interactions, and loops represent autocrine signaling. The number of ligand-receptor interactions is labeled. The dot size reflects the proportional number of cells in each cluster. **C, D** Heatmaps demonstrating dominant outgoing signaling (**C**) and incoming signaling (**D**) patterns for each cell cluster. For the mesenchymal cell cluster, the communication probability (warmer colors show higher probability and cooler colors lower probability) is plotted on the dot plots for the **E** mesenchymal cells as signal senders and **F** mesenchymal cells as signal receivers

The utricle has an epithelial compartment that spans the sensory and non-sensory epithelium (transitional epithelial cells and roof cells), as well as a non-epithelial compartment composed primarily of mesenchymal cells (Figs. 1E and 5A). To test the hypothesis that different cell groups in these compartments are communicating among each other, we utilized the *CellChat* algorithm. This computational algorithm quantitatively infers signaling inputs and outputs using single-cell RNA-seq data [26]. *CellChat* utilizes the Kyoto Encyclopedia of Genes and Genomes (KEGG) database and a manually curated primary literature database to interrogate approximately 2021 ligand-receptor pairs comprising secreted signaling molecules, extracellular matrix-receptor interactions, and cell–cell contacts [26]. We focused our analysis on the five sensory epithelial cell groups and the mesenchymal cells. Our computational data shows a rich environment of intercellular interactions among nearly all the groups of cells tested (Fig. 5B). The cumulative number of significant paracrine signal interactions from the mesenchymal cells totals 63, while roof cells, type II HCs, supporting cells, TECs, and type I hair cells have cumulative significant outgoing signaling interactions totaling 57, 32, 43, 50, and 29, respectively. The number of significant paracrine interactions for mesenchymal cells, roof cells, type II HCs, supporting cells, TECs, and type I HCs were 15, 17, 4, 12, 12, and 1, respectively (Fig. 5B). Taken together, these data reveal the rich paracrine and autocrine signaling that is present among all of the tested cell types with particularly high outgoing signals by the mesenchymal cells.

The identity of the signaling pathways consists of 26 pathways with significant interactions among the different cell types (Fig. 5C and D). These signaling pathways fall into three general outgoing and incoming patterns (Fig. S3). We reasoned that since in multiple other systems mesenchymal cells are dominant signal senders, we focused on mesenchymal cells to identify specific ligand-receptor pairs with statistically significant interactions (Fig. 5E and F) [9, 11, 12]. Different cell groups within the epithelial compartment have varying interactions with the mesenchymal signals that are received (Fig. 5C and D). For example, the IGF pathway through Igf1, Igf2, and Igf3 appears to have ubiquitous but low levels of signaling to all five cell types within the epithelial compartment through both Igf1r and Igf2r. However, Bmp7 signaling that originates from the mesenchyme is primarily received by the roof cells through a combination of co-receptors: Bmpr1b + Bmpr2, Bmpr1a + Bmpr2, and Acvr1 + Bmpr2. Other signaling mechanisms, such as pleiotrophin (PTN), show robust and elevated signal-to-receptor interaction across all the cell types. The PTN pathway stands out with the highest communication probability predicted for mesenchyme as signal senders, while osteopontin (Spp1), pleiotrophin (PTN), and midkine (Mdk) pathways show the highest probability of mesenchymal signal reception (Fig. 5E and F). These data reveal both the heterogeneity and specific patterns of signaling among the cell types of the sensory epithelium and the mesenchyme.

### PTN and TGFβ Signaling

**Fig. 6:**
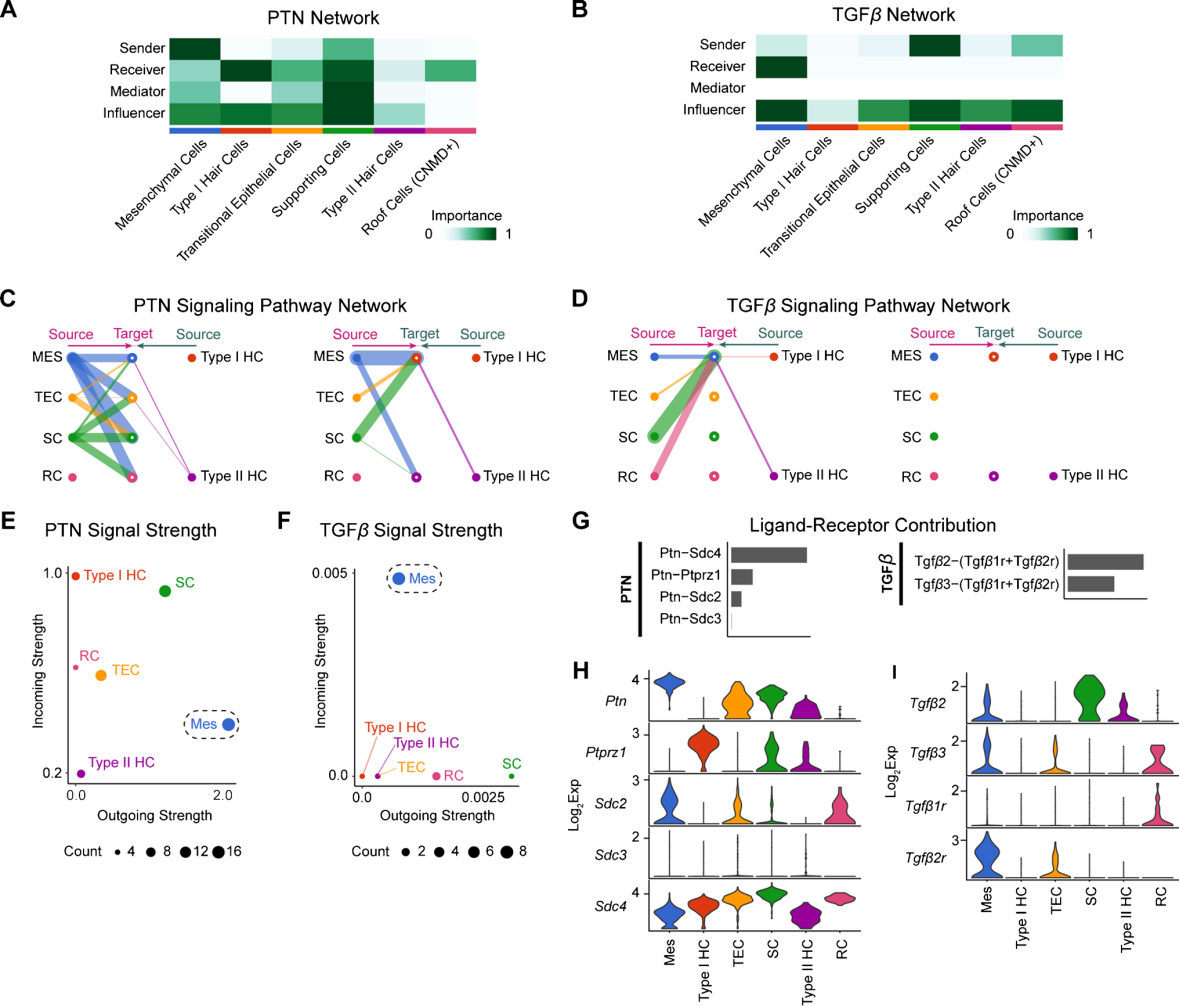
PTN and TGFβ signaling as examples of robust crosstalk between the epithelial and non-epithelial compartments. **A** Social network analysis showing the different network roles (sender, receiver, mediator, or influencer) of each cell cluster in the PTN signaling pathway. **B** Social network analysis showing the different network roles of each cell cluster in the TGFβ signaling pathway. **C** A hierarchy plot highlighting the inferred PTN signaling sources and targets with mesenchyme and supporting cells as the dominant sources of signaling. **D** A hierarchy plot showing the inferred TGFβ signaling sources and targets with supporting cells being the dominant signal senders. **E** The strength of PTN pathway outgoing and incoming signaling for each annotated cell cluster. The mesenchymal cell cluster (Mes) has the highest outgoing signal strength. **F** The strength of TGFβ pathway outgoing and incoming signaling for each annotated cell cluster. The mesenchymal cells display the highest incoming cell signals here. **G** The relative contribution of each ligand-receptor pair for the PTN and TGFβ signaling pathways. **H** Violin plots showing the relative expression of each ligand and receptor in the PTN pathway across annotated cell clusters. **I** Violin plots showing the relative expression of each ligand and receptor in the TGFβ pathway across annotated cell clusters

To further characterize cell–cell interactions between the mesenchymal and epithelial compartments, we focused on two significant developmentally influential pathways: PTN and TGFβ signaling (Fig. 6). The Tgfβ2 signaling pathway has been implicated in early embryonic inner ear development and senescence during the otocyst stage [34]. And similarly, the pleiotrophin pathway has been shown to be involved in cell proliferation and developmental processes in the central nervous system [35, 36]. To determine how a community of cells interacts within the utricle, we applied *CellChat*’s social network analysis tool. Social network analysis determines the role of communication patterns in four categories: sender, receiver, mediator, and influencer [37]. We tested whether we could predict cell group behavior relative to other groups to unveil their communication patterns. Our analysis predicts that pleiotrophin has the highest signal intensity emitted from the mesenchymal cells as senders and type I hair cells as receivers, while supporting cells serve as mediator, influencer, and receiver (Fig. 6A). TGFβ signaling displays more sparse social network signaling with the dominant sending signal coming from supporting cells, while the receivers are predominantly mesenchymal cells (Fig. 6B).

We utilized signaling pathway network analysis to predict which cells were expressing PTN and TGFβ signaling genes (Fig. 6C and D). The source of PTN is predominantly from mesenchyme and supporting cells (Fig. 6C and E). The dominant source of TGFβ signaling is within the epithelial compartment and is comprised mainly of supporting cells and roof cells (Fig. 6D and F). The prime target and signal receivers of TGFβ signaling are the mesenchymal cells (Fig. 6D and F). To assess the relative contribution of different ligand-receptor pairs within a family of genes, we quantified the relative contribution of each ligand-receptor pair in PTN and TGFβ pathways (Fig. 6G–I). Ptn ligand has 4 receptor partners that include Ptprz1, Sdc2, Sdc3, and Sdc4. TGFβ signaling is comprised of Tgfβ2 and Tgfβ3, both bind to receptor pairs Tgfβ1r and Tgfβ2r (Fig. 6G–I). *Ptn* and *Ptprz1* are unique in that there is robust signaling to just the sensory epithelial cells (type I and II hair cells, and supporting cells) that spares the roof cells and TECs (Fig. 6G and H). In practical terms, this analysis reveals that, for PTN signaling, it is likely the mesenchymal cells produce most of the Ptn ligand, and the entire epithelial compartment are the most likely principal receivers. For TGFβ signaling, it is likely that the supporting cells produce the most Tgfβ ligand, and the mesenchyme are the principal receivers. Taken together, these results highlight the complex reciprocal interactions within an organ that go beyond individual cells communicating with each other.

### *In Vivo* Validation of Ligand-Receptor Pairs

**Fig. 7:**
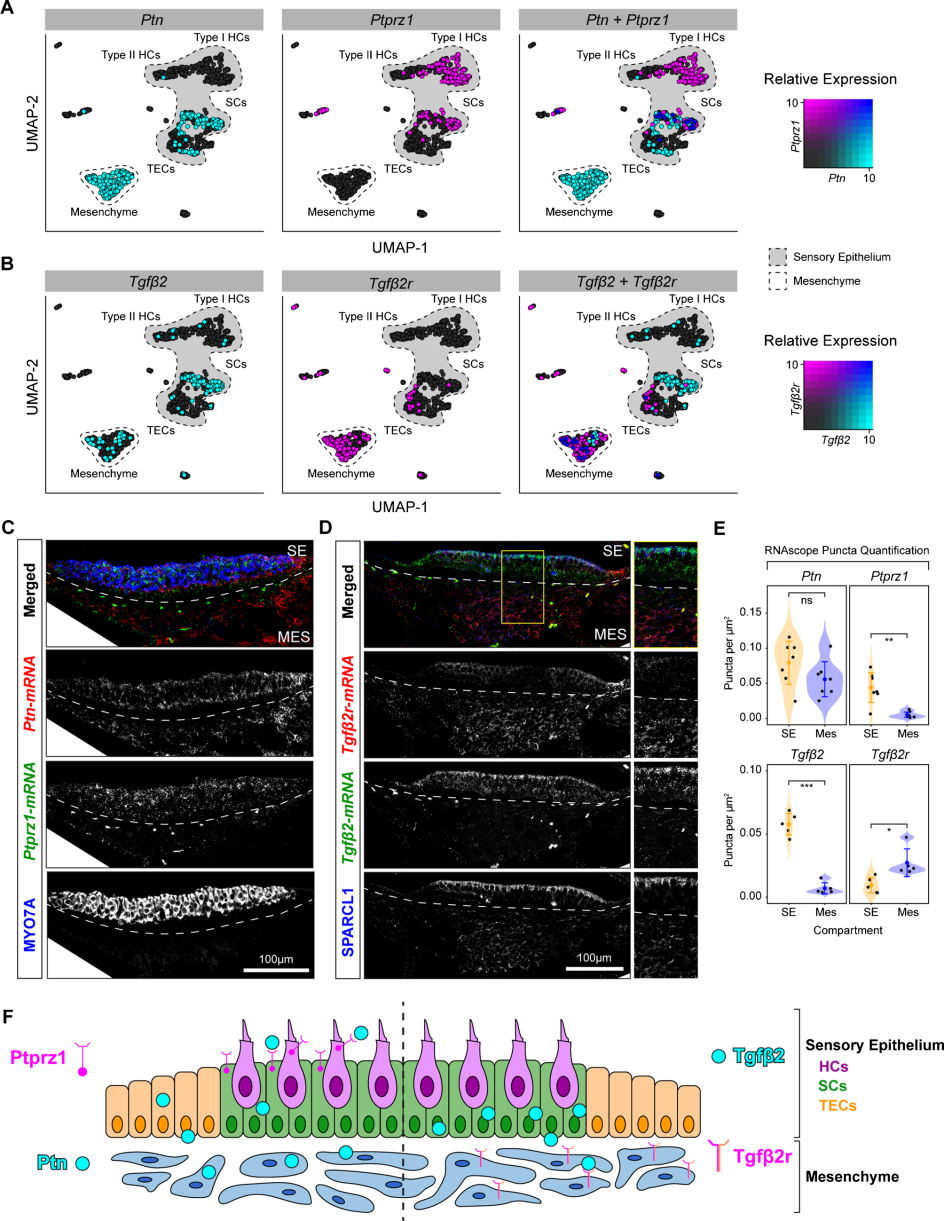
Validation of PTN and TGFβ signaling within the utricle. **A** Co-expression UMAP plot depicting the relative expression of *Ptn* (cyan), *Ptprz1* (magenta), and overlapping expression in blue. *Ptn* expression is primarily in the mesenchyme with some expression in supporting cells that overlaps with *Ptprz1* expression. **B** Co-expression UMAP depicting the relative expression of *Tgfβ2* (cyan), *Tgfβ2r* (magenta), and overlapping expression in blue. There is co-expression of Tgf*β*2r within the mesenchyme but exclusive expression of Tgf*β*2 in the supporting cells. In **A** and **B**, the sensory epithelial cells are outlined and shaded in gray. **C** Cross-section confocal microscopy image depicting the expression of *Ptn* mRNA (red), *Ptprz1* mRNA (green), and hair cells stained with anti-MYO7A antibody (blue). **D** Representative cross-section confocal image depicting the expression of *Tgfβ2* mRNA (green), *Tgfβ2r* mRNA (red), and anti-SPARCL1 antibody (blue). **E** Quantification violin plots of the number of puncta per square micron. The number of puncta per µm^2^ of *Ptn*-mRNA (ligand) expression in the mesenchymal compartment was not statistically significant when compared to the sensory epithelial compartment (*t*-test, *p* = 0.1509). However, *Ptprz1*-mRNA (receptor) expression was high in the sensory epithelium and lower in the mesenchyme (*t*-test, *p* = 0.003095) validating the computational predictions. The number of puncta per µm^2^ of *Tgfβ2-*mRNA (ligand) was also statistically different and was higher in the sensory epithelial compartment when compared to the mesenchymal compartment (*t*-test, *p* = 2.765 × 10e-5). Conversely, the number of puncta per µm^2^ *Tgfβ2r*-mRNA (receptor) was higher in the mesenchymal compartment when compared to the sensory epithelial compartment (*t*-test, *p* = 0.02016). **F** Model showing the roles of Ptprz1/Ptn receptor-ligand combo on the left, and Tgf*β2r*/Tgf*β*2 receptor-ligand on the right

To validate the computational predictions of the cell–cell interactions, we again focused on PTN and TGFβ pathways through in vivo experiments. Analysis of *Ptn* ligand mRNA and *Ptprz1* receptor mRNA distribution in our single-cell data confirms that expression of *Ptn* spans nearly the entire mesenchymal cell population with some expression in the supporting cells and roof cells, while co-expression with *Ptprz1* receptor is restricted primarily to the supporting cells (Fig. 7A). *Ptprz1* receptor is also highly expressed in the hair cells (Fig. 7A). *Tgfβ2* ligand mRNA is primarily expressed in the supporting cells with less expression in the mesenchyme, while *Tgfβ2*-receptor mRNA is mostly expressed in the mesenchyme with minimal expression in the TECs (Fig. 7B). There is minimal overlap in the expression of *Ptn* and *Ptprz1* within the mesenchymal cells (Fig. 7B).

Using combined multiplex in situ hybridization and immunohistochemistry, we validated our computational prediction of the Ptn/Ptprz1 ligand-receptor pair where *Ptn* is nearly ubiquitously expressed throughout both epithelial and mesenchymal compartments, while *Ptprz1* is restricted to the epithelial compartment (Fig. 7C). Quantification of the mRNA abundance levels validates the computational prediction and matches the expression patterns (Fig. 7E). As a second validation target, Tgfβ2 ligand transcript is primarily detected within the epithelial compartment compared to the *Tgfβ2r* transcript that is predominantly expressed in the mesenchymal compartment (Fig. 7D). mRNA puncta quantification shows a statistically significant difference between epithelial and non-epithelial compartments (Fig. 7E). This mRNA expression pattern mimics the predicted expression levels of *Tgfβ2* and *Tgfβ2r* in the single-cell RNA-seq data (Fig. 7B). Taken together, these two receptor-ligand pair patterns of expression validate the computational predictions (Fig. 7F).

### Expression of Hearing Loss and Vestibulopathy Associated Genes

**Fig. 8:**
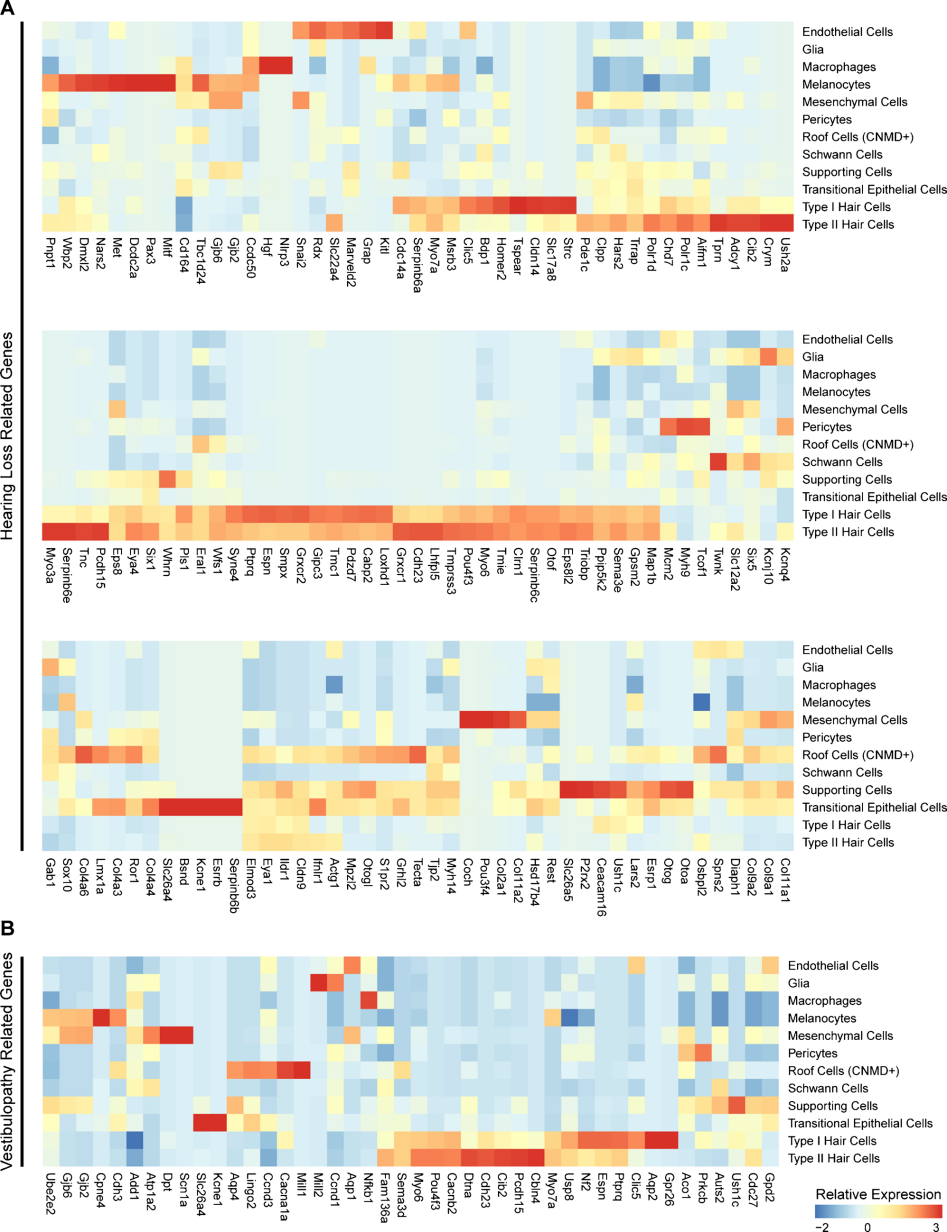
Mapping human hearing loss and vestibular dysfunction-related genes to the annotated mouse utricular single-cell RNA-seq transcriptome. **A** Heatmap of the relative expression of known hearing loss-related genes across the different annotated cell clusters. **B** Heatmap of the relative expression of known vestibulopathy-related genes across the different annotated cell clusters

We wanted to test whether our validated dataset has any relevance to hearing loss-related genes and vestibular disorders. To do this, we used a previously published list of human hearing loss and vestibular disorders associated genes [16]. Not surprisingly, there are many genes in both the hearing loss and vestibular disorders lists that map to hair cells or supporting cells (Fig. 8A and B). However, more interestingly, there are many genes that map to cell groups from the non-epithelial compartment. For example, *Dpt* in mesenchyme, *Cpne4* in melanocytes, *Mill2* in glia. This highlights the importance of non-epithelial cell compartments in health and disease.

## Discussion

In this study, we characterized the transcriptional profile of the postnatal mouse utricle at single-cell resolution. Using carefully curated metadata and the rich transcriptional profile generated by the Smart-seq2 platform, we reveal the heterogeneity of cells within the entire utricle. We tested the hypothesis that the utricular cells interact via a complex array of signaling between the epithelial and non-epithelial compartments.

Not surprisingly, the epithelial compartment, as previously described, contains type I and II hair cells, supporting cells, transitional epithelial cells, and roof cells [4, 5]. Hair cell precursors and subtypes of transitional epithelial cells are not as prominently seen here, likely because of the later time points analyzed here (P4 and P6) than in prior studies that included P1- or P2-derived cells where cell proliferation is more prevalent [4, 5]. The non-epithelial compartment contains seven different cell types. Similar to Wilkerson et al. (2021) [18] in the crista ampullaris, we observed macrophages, endothelial cells, pericytes, melanocytes, glia, and mesenchyme. Macrophages have previously been described within the non-epithelial compartment of the utricle and increase in number within the sensory epithelium following injury [38]. We were further able to detect markers of Schwann cells, but did not observe erythrocytes, likely given the capture technique involving flow cytometry where debris and smaller cells are gated out. The presence of these diverse non-epithelial cell types underscores their importance for homeostasis and postnatal development of the mouse utricle. While the roles of immune cells have begun to be elucidated within the inner ear [39], the roles of other cell types, such as pericytes, warrant further investigation.

Mesenchymal cells in the utricle are the dominant cell group demonstrated by the single-cell RNA-seq data. Within the mesenchyme are the remainder of the non-epithelial cells, as our immunohistochemistry demonstrates the rich vasculature coursing through the tissue (Fig. 3H). Endothelial cells that comprise the vasculature are intimately associated with macrophages as well as pericytes (Fig. 3C, E, and F). This intricate interaction highlights the utricle’s remarkable connection with the immune system, a yet unexplored realm that may lend credence to ideas of immune-mediated inner ear disorders and/or the mysteries of Ménière’s disease. Moreover, the mesenchymal cells primarily resemble two sub-types of cochlear mesenchyme: the lateral wall and spiral limbus (Fig. S2C–D) [17]. The other cochlear mesenchyme subtypes, basement membrane and modiolar mesenchyme, are not well represented in our dataset likely because these structures are more unique to the cochlea.

Our computational analysis predicts a complex array of intercellular communication. Intuitively, this is not surprising; however, the degree of significant communication that is quantifiable is nonetheless remarkable (Fig. 5 and Fig. S3). Aside from the dominance of the mesenchyme as signal mediators, some interesting patterns deserve further attention. For example, supporting cells and transitional epithelial cells are dominant signal senders across the epithelial and non-epithelial compartments. We postulate that supporting cells’ dominance in intercellular signal transduction relates to their multifaceted roles in the development and survival of hair cells and neuronal cells, immune/clean-up function following hair cell death, and as progenitors during development and regeneration [40]. Unsurprisingly, they have been compared to glia in the central nervous system and demonstrate interactions with the glia of the inner ear [41].

The critical role of mesenchymal cells has previously been shown in vitro for organoid differentiation protocols of the inner ear; however, the molecular mechanisms of this process have remained elusive [42]. Our hypothesis that mesenchymal cells are the dominant signal senders is borne out by both paracrine and autocrine signaling strengths. We first focused on the pleiotrophin pathway, specifically the Ptprz1 receptor, because of its recent significance within the neuroscience literature linking it to neuronal differentiation [43]. Pleiotrophin is a heparin-binding cytokine [44]. Hippocampal neurogenesis has been linked to the Ptn-Ptprz1 pathway through Akt signaling [45]. Our transcriptional atlas identified this mechanism with robust signaling among the different compartments, and validation experiments show the prevalence of the Ptprz1 receptor mRNA within the sensory epithelium. This pathway is ripe for further investigation in the inner ear and manipulation both at the neonatal stages and in adults, with a focus on proliferation.

The second receptor-ligand pair we validated is from the transforming growth factor-β (TGFβ) family of receptors. TGFβ family members function as homodimers or heterodimers and are known to stimulate proliferation and differentiation during development across multiple cell lineages [46]. TGFβ receptors are classified as serine-threonine kinases with two major receptor types: I and II (TGFβ1-receptor and TGFβ2-receptor). With ligand binding, heteromeric receptor complex formation and stabilization leads to intracellular signaling cascade [46]. Our dataset shows a significant expression of *Tgfβ2* mRNA, which encodes the TGFβ ligand in the supporting cells and type II hair cells, as well as a modicum of expression from the mesenchyme. The receptors involve heterodimerization of the Tgfβ1-receptor and Tgfβ2-receptor (Fig. 6G). The highest signaling strength to the mesenchyme (Fig. 6F) supports the notion of epithelial to mesenchyme communication and its role during early postnatal development. Within the inner ear, Tgfβ1-receptor activity is known to be required for lateral prosensory domain formation during cochlear embryonic development [47]. Taken together, TGFβ signaling is a critical component of the cell–cell communication between different compartments of the utricle.

In sum, our study provides an in-depth transcriptional profile of the postnatal mouse utricle with a focus on the non-epithelial compartment. We highlight the diverse array of cell–cell communication patterns among specific cell types and between cellular compartments. Notably, the mesenchymal cells are the dominant signal senders during early postnatal development of the utricle in the non-epithelial compartment. Our quantitative and validated dataset of receptor-ligand expression patterns within the postnatal utricle serves as a resource for the scientific community.

## Limitations of the Study

There are two main limitations of this study: First, the number of sequenced cells for single-cell RNA-seq is on the lower side considering some of the droplet-based technologies that contain thousands of cells. This for example may have limited some of our predictive power when comparing to the existing cochlear mesenchyme dataset [17]. However, our study does provide high-quality full-length Smart-seq2 protocol sequencing which mitigates some of these issues. In the future, more cells will need to be added to study the non-mesenchymal cells within the non-epithelial compartment that had too few cells for more in-depth analysis such as the melanocytes identified. Second, while we validated at the mRNA level two receptor-ligand pairs, future studies would take this further by interrogating these pathways using agonist/antagonist in vitro experiments.

## Supplementary Information

Below is the link to the electronic supplementary material.

Supplementary file1 (XLSX 576 KB)

Supplementary file2 (XLSX 823 KB)

Supplementary file3 (XLSX 20 KB)

Supplementary file4 (DOCX 3951 KB)
