# Supplementary material for: Crosstalk Signaling Between the Epithelial and Non-Epithelial Compartments of the Mouse Inner Ear
Source: J Assoc Res Otolaryngol. 2025 Mar 13;26(2):127–45. doi: 10.1007/s10162-025-00980-7 (PMC11996748; doi:10.1007/s10162-025-00980-7)
Supplement: Supplementary file 4 — Supplementary file4 (DOCX 3951 KB) [file 10162_2025_980_MOESM4_ESM.docx]

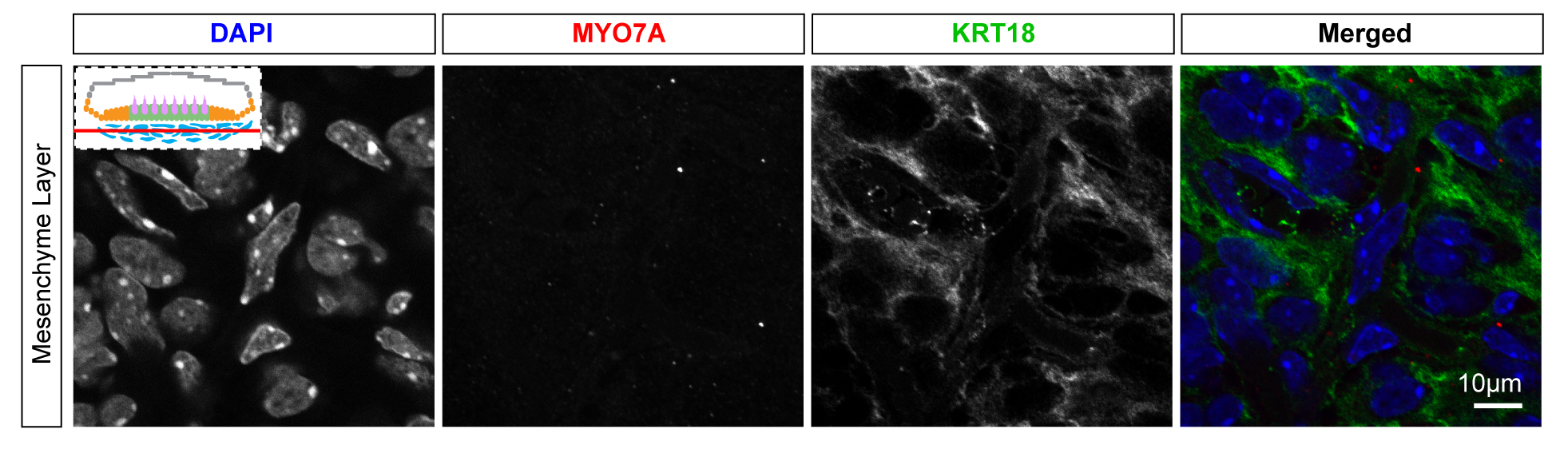


**Fig. S1** Whole mount utricle with anti-KRT18 antibody (green) labeling at the mesenchymal layers (same specimen as Fig 2G). This shows minimal KRT18 staining at this layer. Inset of cartoon diagram indicating the level of the image (red horizontal line).


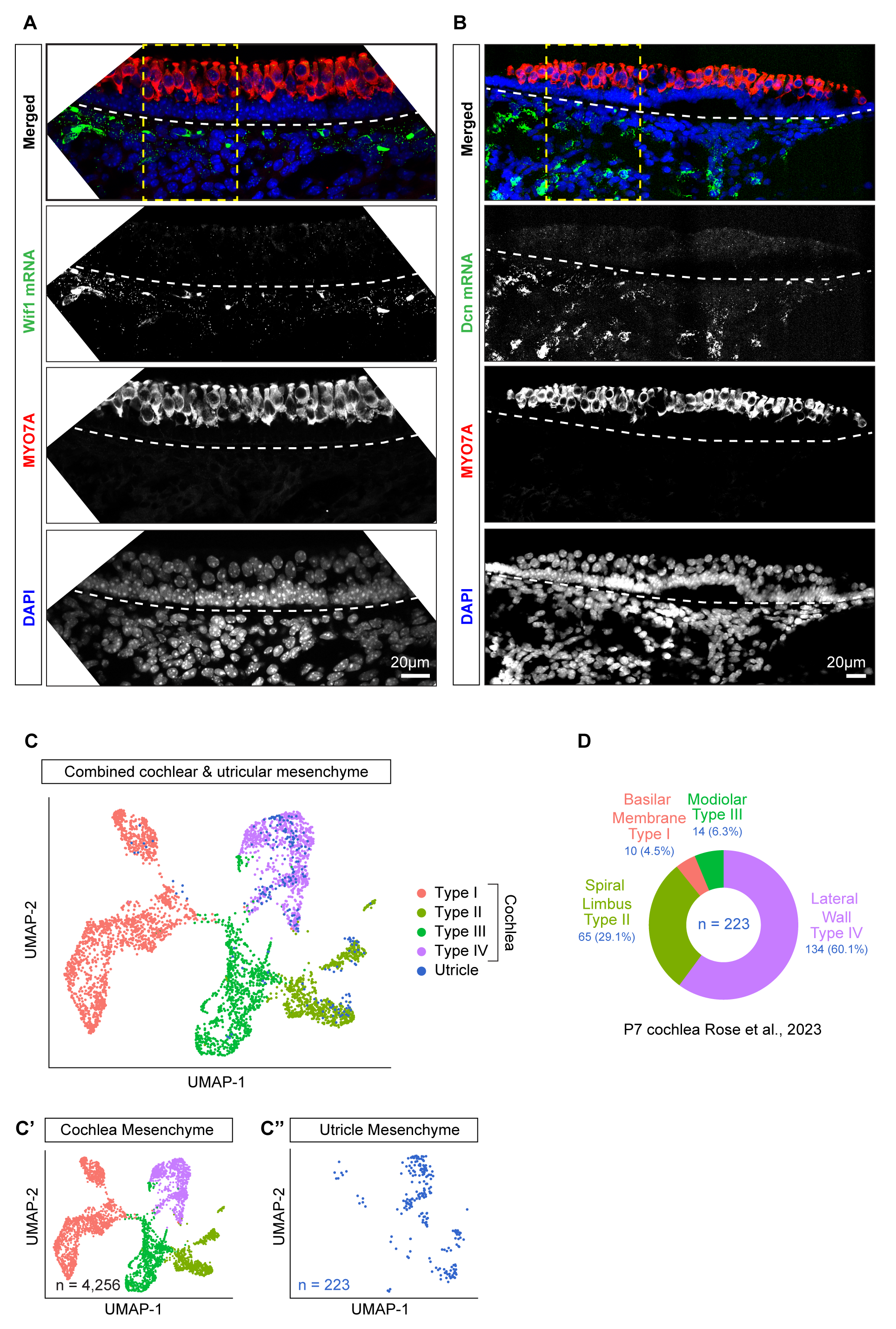


**Fig. S2** (A-B) Cryosection confocal image of combined immunohistochemistry and *in situ* hybridization. (A) *Wif1* mRNA (green), anti-MYO7A (red), and DAPI nuclei (blue) shows primarily expression of *Wif1* in the mesenchymal layer. Insets from this exact image are shown in Fig 4I’. (B) *Dcn* mRNA (red), anti-MYO7A (green), and DAPI nuclei (blue) highlight expression of *Dcn* primarily in the mesenchymal layer. Insets from this exact image (yellow dashed boxes) are shown in main Figure 4J’. (C) Combined UMAP of P7 cochlear mesenchyme from Rose et al. (2023) and P4 and P6 utricular mesenchyme from our dataset. Note the four sub-types of cochlear mesenchyme are basilar membrane (type I), spiral limbus (type II), modiolar (type III), and lateral wall (type IV). The utricle mesenchyme primarily co-cluster with the type II and IV cochlear mesenchyme sub-types (C’-C’’). (D) Sunburst plot showing quantification of the co-clustering frequency comparing the 223 utricular mesenchyme cells relative to the cochlear mesenchyme subtypes. Over half of the utricular mesenchyme co-cluster with the lateral wall type IV cochlear mesenchymal cell types.


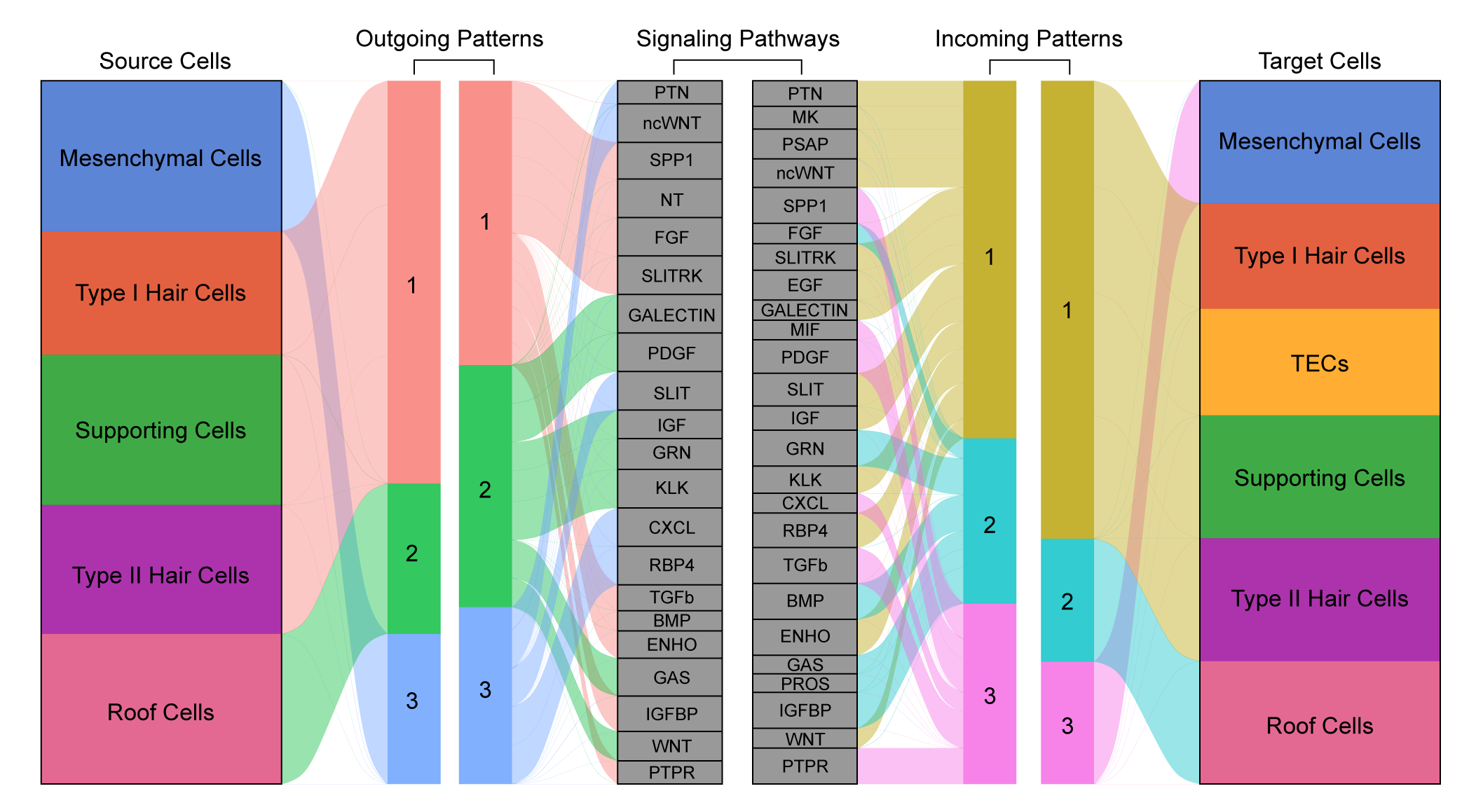


**Fig. S3** River plot depicting three computationally determined patterns of outgoing signaling and their corresponding pathways on the left. On the right, are the three computationally determined patterns of incoming signaling patterns and their corresponding pathways.
